# A Rare Side Effect due to TNF-Alpha Blocking Agent: Acute Pleuropericarditis with Adalimumab

**DOI:** 10.1155/2013/985914

**Published:** 2013-07-18

**Authors:** Hakan Ozkan, Ahmet Seckin Cetinkaya, Tekin Yildiz, Tahsin Bozat

**Affiliations:** ^1^Department of Cardiology, Medical Park Hospital, Hasim Iscan Cad, Fomara Meydani, No. 1, Osmangazi, 16220 Bursa, Turkey; ^2^Department of Respiratory Disease, Medical Park Hospital, 16220 Bursa, Turkey

## Abstract

Tumor necrosis factor-alpha antagonism is an important treatment strategy in patients with rheumatoid arthritis, psoriatic arthritis, vasculitis, and ankylosing spondylitis. Adalimumab is one of the well-known tumor necrosis factor-alpha blocking agents. There are several side effects reported in patients with adalimumab therapy. Cardiac side effects of adalimumab are rare. Only a few cardiac side effects were reported. A 61-year-old man treated with adalimumab for the last 6 months due to psoriatic arthritis presented with typically acute pleuropericarditis. Chest X-ray and echocardiography demonstrated marked pericardial effusion. Patient was successfully evaluated for the etiology of acute pleuro-pericarditis. Every etiology was excluded except the usage of adalimumab. Adalimumab was discontinued, and patient was treated with 1200 mg of ibuprofen daily. Control chest X-ray and echocardiography after three weeks demonstrated complete resolution of both pleural and pericardial effusions. This case clearly demonstrated the acute onset of pericarditis with adalimumab usage. Acute pericarditis and pericardial effusion should be kept in mind in patients with adalimumab treatment.

## 1. Introduction

Tumor necrosis factor-alpha (TNF*α*) antagonism is an important treatment strategy in patients with rheumatoid arthritis, psoriatic arthritis, vasculitis, and ankylosing spondylitis [1, 2]. Infliximab, etanercept, and adalimumab are indicated in immune-mediated inflammatory diseases. Several side effects including congestive heart failure, skin disorders, tuberculosis, and malignancy could be seen during the treatment [3].

## 2. Case

**Figure 1:**
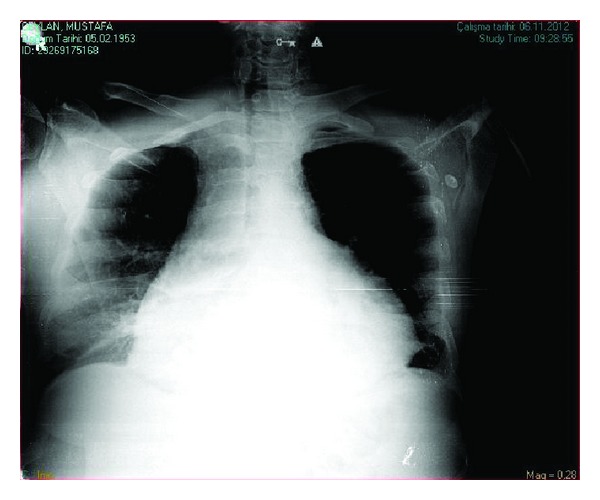
Chest X-ray demonstrating cardiomegaly with pericardial effusion.

**Figure 2:**
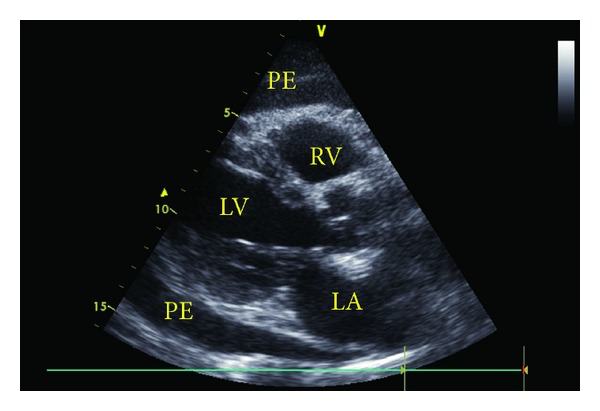
Parasternal long axis view with transthoracic echocardiography demonstrating pericardial effusion (PE: pericardial effusion, RV: right ventricle, LV: left ventricle, and LA: left atrium).

**Figure 3:**
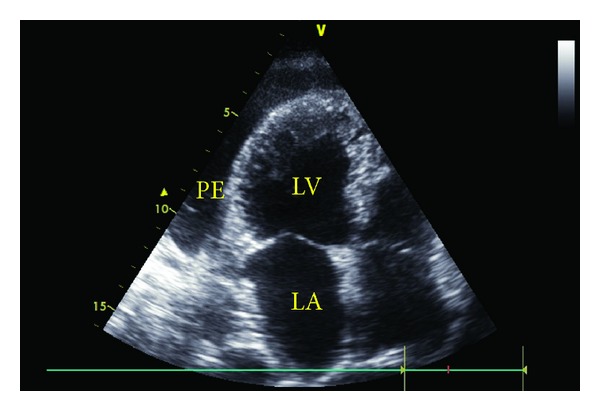
Apical four-chamber view with transthoracic echocardiography (PE: pericardial effusion, LV: left ventricle, and LA: left atrium).

A 61-year-old man treated with adalimumab for the last 6 months due to psoriatic arthritis presented with sudden onset of chest pain, shortness of breath, palpitation, cough, and signs of right heart failure. Physical examination revealed bilaterally bibasilar fine crackles with ortopnea and tachypnea. Heart sounds were markedly decreased with pericardial frotman. Chest X-ray showed significant cardiomegaly and bilateral pleural effusion (Figure 1). The patient underwent to transthoracic echocardiography. Echocardiographic examination showed moderate to large pericardial effusion without cardiac tamponade (Figures 2 and 3). Etiology causing acute pleuropericarditis was carefully evaluated. 

The laboratory findings were as follows: Hb 11.2 g/dL, WBC 5.36 K/uL, PLT 319 k/uL, CRP 157 mg/L, erythrocyte sedimentation rate (ESR) 87 mm/h, TSH 0.75 mIU/L, and plasma antinuclear antibody (ANA) level was negative.

**Figure 4:**
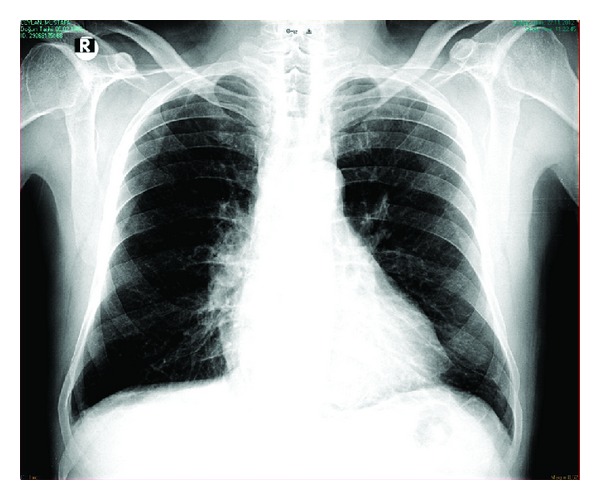
Chest X-ray after three weeks. Complete resolution of pericardial and pleural effusions.

We thought that adalimumab was the cause of the acute pleuro-pericarditis. Adalimumab discontinued and treated with ibuprofen 1200 mg daily. Patient was discharged after a four-day treatment. Control chest X-ray and echocardiography after three weeks demonstrated complete resolution of both pleural and pericardial effusions (Figure 4).

## 3. Discussion

There are several reasons that result into resulting pleuropericarditis. Uremia [4], inflammatory bowel disease [5], systemic lupus, drug induced lupus erythematosus, rheumatoid arthritis, and infections may cause pleuropericarditis [6]. Pericardial reactions to drugs are rare. Hiz et al. reported pleuropericarditis with sulphasalazine [7]. TNF-alpha blockers such as adalimumab may cause hypertension, heart failure, atrial fibrillation. However, pericarditis, pericardial effusion, and lupus-like syndrome are rare side effects of adalimumab. Soh et al. reported large pericardial effusion and cardiac tamponade in two romatoid artrit patients who were treated with adalimumab [8]. Both of them underwent surgery. 

In our case, there were signs of pleuropericardial effusion along with sudden onset of chest pain, shortness of breath, palpitation, cough, and signs of right heart failure. We treated our patient with a discontinuation of adalimumab and daily 1200 mg a ibuprofen of clinical findings dramatically improved within days, and complete resolution occured after three weeks.

In conclusion, when symptoms of pleuropericarditis occur in a patient with receiving adalimumab, it should be kept in mind that cardiopulmonary involvement might be associated with adalimumab and medication should be discontinued immediately.
